# Optimizing the Performance of the Sparse Matrix–Vector Multiplication Kernel in FPGA Guided by the Roofline Model

**DOI:** 10.3390/mi14112030

**Published:** 2023-10-31

**Authors:** Federico Favaro, Ernesto Dufrechou, Juan P. Oliver, Pablo Ezzatti

**Affiliations:** 1 Instituto de Ingeniería Eléctrica, Facultad de Ingeniería, Universidad de la República, Montevideo 11300, Uruguay; jpo@fing.edu.uy; 2 Instituto de Computación, Facultad de Ingeniería, Universidad de la República, Montevideo 11300, Uruguay; edufrechou@fing.edu.uy (E.D.); pezzatti@fing.edu.uy (P.E.)

**Keywords:** sparse NLA, FPGA, energy consumption, performance modeling

## Abstract

The widespread adoption of massively parallel processors over the past decade has fundamentally transformed the landscape of high-performance computing hardware. This revolution has recently driven the advancement of FPGAs, which are emerging as an attractive alternative to power-hungry many-core devices in a world increasingly concerned with energy consumption. Consequently, numerous recent studies have focused on implementing efficient dense and sparse numerical linear algebra (NLA) kernels on FPGAs. To maximize the efficiency of these kernels, a key aspect is the exploration of analytical tools to comprehend the performance of the developments and guide the optimization process. In this regard, the roofline model (RLM) is a well-known graphical tool that facilitates the analysis of computational performance and identifies the primary bottlenecks of a specific software when executed on a particular hardware platform. Our previous efforts advanced in developing efficient implementations of the sparse matrix–vector multiplication (SpMV) for FPGAs, considering both speed and energy consumption. In this work, we propose an extension of the RLM that enables optimizing runtime and energy consumption for NLA kernels based on sparse blocked storage formats on FPGAs. To test the power of this tool, we use it to extend our previous SpMV kernels by leveraging a block-sparse storage format that enables more efficient data access.

## 1. Introduction

The dissemination of massively parallel processors in the last decade irreversibly changed the landscape of high-performance computing (HPC) hardware. Recently, this true revolution has propelled the development of field-programmable gate arrays (FPGAs) as hardware accelerators for HPC applications. Consequently, FPGAs are becoming an appealing alternative to more power-hungry many-core devices in a world increasingly preoccupied with energy consumption [1,2]. One of the key restrictions on the widespread adoption of FPGAs in the software community is their programming model, which is radically different from standard software languages. However, manufacturers have been working to adopt high-level synthesis (HLS) languages like System C, C/C++, or OpenCL to facilitate development and enhance productivity. A remarkable example of this policy is the introduction of SDKs for HLS by the largest FPGA manufacturers, Intel and AMD-Xilinx. Another indicator is the increasing number of research works in HLS [3] and the emergence of multiple HLS tools.

The new developments in the programmability of FPGAs allow these devices to abandon their original niche and be used to solve general-purpose problems. In particular, several recent works on FPGAs focus on the implementation of numerical linear algebra (NLA) kernels [4,5,6], which are a crucial component of a myriad of applications. However, most works focus on dense NLA [7,8,9], as well as other algorithms more suited for FPGAs such as image processing [10,11], machine learning [12], etc. Sparse linear algebra, on the other hand, poses several challenges due to its irregular access patterns and low computational intensity. In this sense, we advanced in developing efficient implementations of the sparse matrix–vector multiplication (SpMv) kernel for FPGAs, both from the viewpoint of speed and energy consumption. In [13], we developed basic SpMv kernels and worked on optimizing their energy consumption. Additionally, we experimentally studied the performance roof of our kernels and included some techniques to overcome the main bottlenecks in [14].

Besides the empirical study, it is also interesting to apply analytical tools to understand our developments’ performance and guide these kernels’ optimization process. Several research works focus on developing models and frameworks to estimate the performance of FPGA designs [15,16,17]. In this sense, the roofline model (RLM) [18] is a graphical tool that can help analyze the computational performance and the main bottlenecks of a specified software when it runs in a particular hardware platform. Specifically, the RLM is a chart that relates the computational performance (usually measured in floating-point operations per second, FLOPS) with the arithmetic intensity (the ratio of floating-point operations per byte of memory transferred). The chart also shows the memory bandwidth and arithmetic performance bounds of the hardware.

The main contribution of this work is an extension of the RLM that allows the optimization of performance and energy consumption of NLA kernels based on sparse block format storage on FPGAs. The model works on operations per memory access instead of the traditional computational intensity and features a quick mechanism to find the performance and energy efficiency of sparse algebra kernels that use sparse block representation formats. As a minor contribution, we present a SpMv kernel HLS implementation that exploits a block-sparse storage format to enable more efficient data access, which we use to put our RLM model to test.

The rest of the paper is structured as follows. In Section 2, we present the SpMv kernel and related works of its implementation in FPGAs as well as the main concepts of the RLM, with a special focus on the main literature about its use with FPGAs. Next, in Section 3, we describe our previous efforts in developing SpMv kernels in FPGA and the new designs for this kernel, and we leverage the RLM to offer a visual procedure to improve, from a runtime and energy consumption perspective, our SpMv method. The experimental evaluation follows this in Section 4. Finally, Section 5 contains the concluding remarks and an outline of future research.

## 2. The **SpMv** and the Roofline Model

In this section, we present the SpMv kernel and the works related to its implementation in FPGAs. Afterward, we introduce the roofline model and discuss related works on its utilization in FPGAs.

### 2.1. The SpMv Kernel

Multiplying a sparse matrix by a vector (SpMv) is an essential operation of numerical linear algebra. In many scientific problems, it is one of the most resource-consuming stages. The most typical example is the solution of linear systems of equations using Krylov subspace methods. Such methods imply the repeated use of the SpMv kernel, keeping the same (sparse) matrix but varying the dense vector.

The importance of this kernel has motivated considerable efforts focused on its optimization or performance improvement. These efforts have also accompanied the historical evolution of HPC platforms, and therefore, it is possible to find efficient implementations of the SpMv for the most widespread hardware platforms. However, as this evolution is constant, new implementations and parallel algorithms for the SpMv are still being proposed. In Section 2.2, we present related works about efficient implementations of SpMv in FPGAs.

Algorithm 1 summarizes the serial version of the SpMv, where the sparse matrix *A* is stored in compressed sparse row (CSR) format [19].The non-zero elements are stored in vector val, the column index of each element in the matrix *A* is stored in a vector col_idx, and row_ptr stores the index of the first element for each row in vector val. The non-zero elements within each row are arranged by column index.
**Algorithm 1** Serially computed sparse matrix–vector multiplication (SpMv) with the sparse matrix *A* stored in CSR format.**Input:** 
row_ptr,col_idx,val,x **Output:** 
*y*
 1:y=0 2:**for** i=0 **to** n−1 **do** 3:       **for** j=row_ptr[i] **to** row_ptr[i+1]−1 **do** 4:            y[i]=y[i]+val[j]·x[col_idx[j]] 5:       **end for** 6:**end for**


The main approach to parallelizing this operation using the CSR format is to exploit the absence of data dependencies between the computations associated with each row. However, this approach presents severe load imbalances (depending on the sparsity pattern) and suffers from indirect data access to the dense vector *x*.

### 2.2. SpMv in FPGA

In [20], the authors present an architecture for the SpMv that can process non-zero elements from multiple rows in parallel. They introduce a variation of the CSR matrix representation format called CSR Interleaved to achieve this. Furthermore, they eliminate the necessity of maintaining separate copies of the vector *x* for each multiplier by introducing the concept of a banked vector buffer (BVB). In this approach, the vector *x* is divided into 32 distinct memory banks, and a crossbar is utilized to direct non-zero elements to their respective vector banks.

In [21], the authors develop a streaming dataflow architecture for the SpMv operation and test it in a Xilinx ZynqMP FPGA standalone board. Their proposed solution features a deep pipeline that continuously consumes input data without stalling. To add parallelism, they simultaneously process multiple rows in separate compute pipelines, each with its own I/O ports. This requires merging row and column indices within the same input port due to the limited number of I/O ports available on the device.

The authors in [22] analyze the performance and energy efficiency of SpMv and other kernels in two FPGA platforms, one with DDR memory and the other with HBM, and compare the results with multi-core CPUs and a GPU. They include the empirical roofline model (using the Empirical Roofline Toolkit extended for FPGAs) to enhance the platform characterization and cross-platform comparison. They find that FPGAs are well behind GPUs in absolute terms for computing and memory-intensive kernels but require far less power and are competitive in energy efficiency.

Yixiao Du et al. [23] developed HiSparse, a high-performance kernel for HBM-equipped FPGAs. HiSparse uses a custom format for storing sparse matrices in HBM, allowing vectorized streaming access to each HBM channel and concurrent access to multiple channels. This helps to fully utilize the available bandwidth of HBM for loading the sparse matrix. In addition, HiSparse features a scalable on-chip buffer design that incorporates vector replication and banking to support a large number of parallel processing engines (PEs). This helps to maximize data reuse in accessing the input vector. HiSparse can support arbitrarily large matrices through the use of matrix partitioning along both rows and columns.

Xilinx offers its own set of open-source HLS libraries known as Vitis libraries, which include one for the BLAS specification and another for sparse operations [24]. These libraries provide optimized blocks for sparse data movement and calculations, as well as an SpMv kernel that leverages these blocks. The SpMv kernel provided by the library is specifically optimized for Xilinx’s Alveo architectures, efficiently utilizing HBM memory and a matrix partitioning scheme. To our knowledge, this represents the most high-performing SpMv implementation on Alveo platforms. It is worth noting that the objective of our work is not to deliver the best performance implementation but rather to present it as a case study to demonstrate the application of the proposed RLM techniques on FPGAs.

### 2.3. Roofline Model (RLM)

The roofline model (RLM) is a graphical tool that provides performance estimates for a computational kernel running on a specific hardware platform. It is particularly valuable for identifying performance bottlenecks and offering optimization guidance, helping programmers and architecture designers achieve maximum computational efficiency.

At its core, the roofline model combines three key concepts within a single plot: computational peak performance (CP) measured in GFLOP/s, which represents the maximum number of floating-point arithmetic operations that can be executed per second, memory bandwidth (BW) measured in GB/s, that quantifies the maximum rate at which data can be transferred between off-chip memory and computational units, and computational intensity (CI), which measures the extent of data reuse between off-chip memory and computational units. Computational intensity is often calculated as the ratio of useful operations performed to the amount of memory transferred to and from off-chip memory in a given computational kernel.

**Figure 1:**
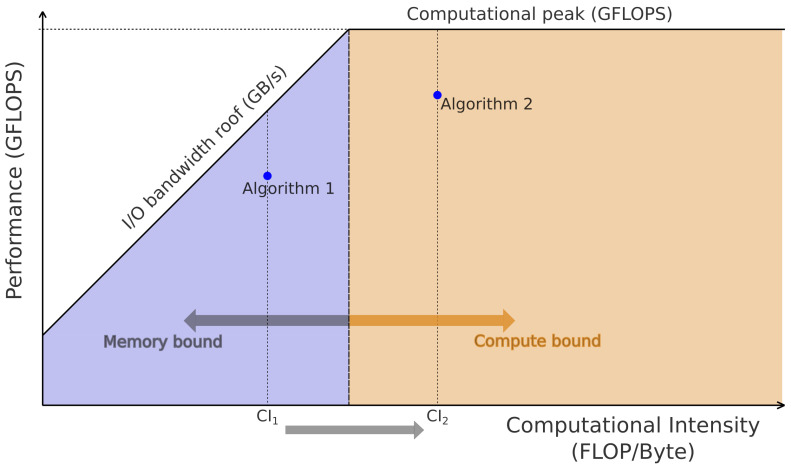
Roofline Model graph.

Figure 1 summarizes the fundamental concepts of the model, displaying the performance ceiling in GFLOPS and the bandwidth ceiling in GB/s. The point of intersection between peak bandwidth and peak performance is referred to as the ridge point. Applications located to the left of this ridge point are considered memory-bound, indicating that their performance is primarily constrained by memory bandwidth. Conversely, applications to the right of the ridge point are classified as compute-bound, implying that the computational resources of the hardware platform predominantly limit their performance. In Figure 1, Algorithm 1 has a low CI, which causes it to be limited by bandwidth. As the CI increases, Algorithm 2 becomes compute-bound.

When employing the roofline model with FPGAs, some considerations should be considered. Firstly, it is important to note that FPGAs lack a fixed architecture; their architecture is determined by the algorithm in use. As a result, the performance ceiling depends on the specific algorithm employed. While it is possible to establish an absolute performance limit based on the hardware resources available for arithmetic operations, this value may deviate significantly from reality depending on the chosen algorithm. Additionally, it is crucial to recognize that some resources are used for routing, and the clock frequency is also influenced by the design. On a positive note, in FPGAs, the designer has complete control over the management of internal (fast) memories, which allows them to precisely determine the number of memory accesses at all levels. This makes it possible to accurately calculate computational intensity, unlike in CPU architectures, where obtaining this value can be challenging because of the hard-to-predict cache-access patterns.

### 2.4. RLM in FPGAs

Although the roofline model is not completely widespread in the FPGA community, some interesting published works can be found. Da Silva et al. [25] proposed using the RLM for each algorithm optimization in FPGA with high-level synthesis tools. The authors employed different metrics, expressing the computational intensity as ByteOperations over ByteMemoryAccess, and the performance as ByteOperations per second.

M. Pahlavan [26] adapted the RLM for FPGAs in his master’s thesis. Pahlavan proposed the FPGA-Roofline, focusing on defining the bandwidth link as the main bottleneck and representing the available throughput (operations per second) per operational intensity.

E. Calore [27] introduced the FPGA empirical roofline (FER), which serves as a benchmarking instrument for empirically assessing computing performance and external memory bandwidth of FPGAs in HPC applications. Their approach allows cross-architectural comparisons and provides an estimation of the performance potential for kernels developed using HLS tools according to their arithmetic intensity.

I. Panagou [28] proposed an application-centric roofline model that takes into account both resource limitations and latency constraints, attaining a more precise Roofline ceiling. They expanded this model to support multiple kernels implemented within the FPGA fabric and on the CPU in MPSoC platforms. Additionally, considering that the arithmetic intensity may vary across various inputs, they proposed an input-driven model that enables better optimization strategies.

Recently, D. Diakite et al. [29] leveraged the RLM to optimize a tomography application in OpenCL in an FPGA. The authors defined the performance roof as the number of resources consumed and the effective operation frequency, and the graph employed the same axis as M. Pahlavan’s proposal.

T. Nguyen et al. [30] deeply explored RLM use in the FPGA context. Additionally, their effort addressed the energy consumption problem and the SpMv kernel. M. Siracusa et al. [31] extended this work by defining a comprehensive methodology to optimize FPGA designs based on the RLM.

## 3. Implemented Kernel and RLM

In this section, we first present the SpMv kernel implementation. Next, we explain how we use the roofline model to improve the kernel implementation, and finally, we incorporate the analysis of energy consumption into the model.

### 3.1. Implemented Kernel

In a previous effort, we developed an FPGA kernel to compute SpMv operation in CSR format. The kernel proceeds row-wise and, to alleviate the indirect accesses to vector *x*, the vector is cached entirely in the FPGA’s internal memory. We opted for a coarse-grained parallelism approach to optimize the kernel, instantiating it multiple times (Compute Unit Replication). However, as this also implies the replication of the *x* cache, the number of CUs is severely limited by the FPGA resources.

In a posterior effort, we explored fine-grained parallelism (i.e., vectorizing the kernel’s data path), requiring a completely different kernel architecture and sparse representation formats.

One of the main obstacles in implementing the SpMv in FPGA is that the floating-point addition has considerable latency. Moreover, the product and accumulation of the partial results (Line 4 in Algorithm 1) presents a data dependency between loop iterations. This dependency prevents the operation from producing one result per clock cycle (i.e., pipeline parallelism). A method to “hide” this data dependency is to interleave the processing of *L* rows simultaneously, where *L* is the latency of the floating-point addition.

For this strategy to be efficient, data must be interleaved before they are read by the FPGA, allowing contiguous memory access. To facilitate this, we opted for the sliced ELL format, which packs the non-zero elements (and their indices), creating dense blocks. We divide the matrix into partitions of *L* rows and then apply ELL to each partition. The longest row determines the width of each block, padding the rest of the rows with zeros to match its length. Then, the blocks are stored in memory so that the *L* rows are interleaved (blocks are transposed). We refer to the implemented kernel architecture that uses this format as *Scalar*.

**Figure 2:**
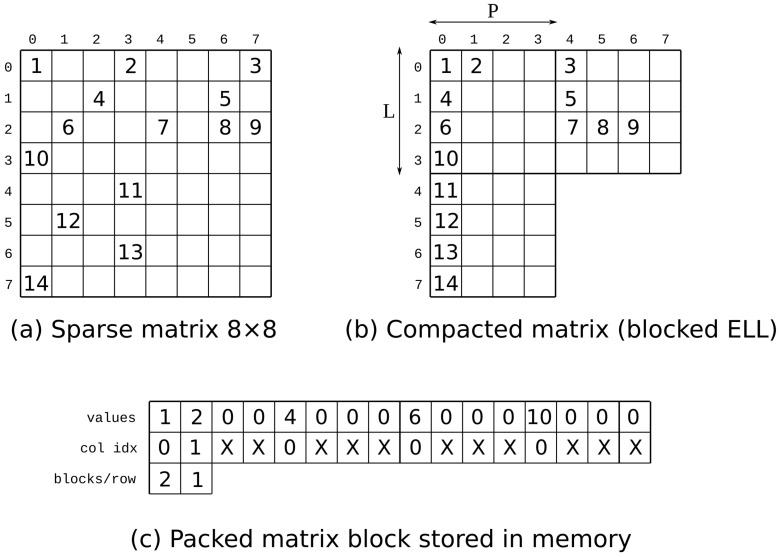
Example of the blocked ELLPACK sparse format with a 8 × 8 matrix, L=4 and P=4.

The issue with this format is that when trying to vectorize the kernel, non-contiguous elements of the vector *x* are simultaneously accessed. The previous format is extended to address this problem so that the blocks have a fixed width *P* (with ideas similar to the SELL-P sparse storage format [32]). This way, the elements of *x* for each block do not have a distance greater than *P*. Then, we can partition the *x* cache in the FPGA to allow *P* simultaneous accesses. We show an example of the sparse format in Figure 2. In addition to the data and column indices, the format requires a vector indicating the number of dense blocks per row of blocks. We refer to this format as Blocked-ELL and to the implemented kernel architecture that uses this format as *Block*.

**Figure 3:**
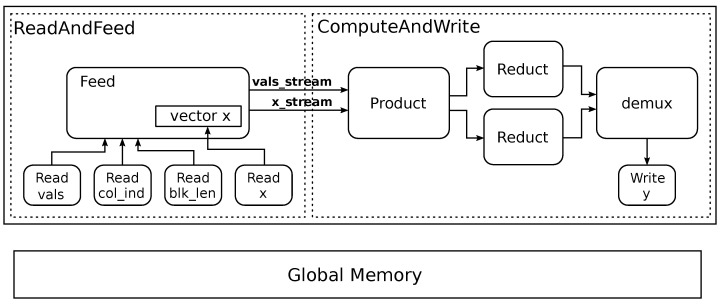
Block diagram of the implemented SpMv kernel with fine-grained parallelism.

A diagram of the resulting kernel, which was implemented using Xilinx’s HLS tools (Vitis 2020.2), is shown in Figure 3. The architecture consists of two parts. The first, on the left, is in charge of reading the data from memory and arranging it before it is passed to the second part, which performs the computations. All the blocks are instantiated in parallel, work simultaneously (i.e., dataflow operation), and connect by FIFO channels. The datapath has a width of *P* elements (floats for values and *x*, and int for indices). The computation block has two available reducers and switches between them to avoid delays.

### 3.2. The RLM as a Guide to Optimizing the Performance of Blockwise SpMv

The reviewed works in Section 2.4 provide detailed information about employing the RLM in optimizing the SpMv for implementations where the operation count and data transfers can be directly derived from the number of non-zeros of the sparse matrix. However, these models are inaccurate for sparse formats such as ELLPACK, where a certain amount of zero-padding is allowed to improve memory performance. Therefore, we propose an adaptation of the RLM that considers the zero-padding to study the bottlenecks in our sparse matrix kernels. In other words, we offer a methodology to leverage the RLM to optimize block-sparse codes in FPGAs, where the key is the procedure to increase the computational intensity when the kernel is in the memory-bound region (blue zone in Figure 1). The advantage of the RLM is that it provides straightforward visual verification of the procedure. Some related ideas were previously presented for dense algebra methods running in GPUs [33].

We define the model’s vertical axis as performance in GFLOPs and the horizontal axis as CIA (computational intensity per access), representing floating-point operations per memory access. For sparse blocked formats, this metric is related to the density of the blocks. With this definition of CIA, the bandwidth roof is expressed in giga memory accesses per second. Consequently, an increase in the operation count due to a rising amount of data transferred in each memory access impacts the intensity but not the bandwidth.

Sparse codes that employ zero-padded blocks reach two different intensities: the theoretical intensity, defined by the block’s dimension, and the useful intensity, which only considers the number of non-zero coefficients in each block. We employ the second one, computed using the average of non-zero coefficients per block, since counting floating-point operations performed with zeros artificially increases computational intensity and performance. Therefore, the CIA reached by the SpMv in our model depends on the block dimension and the sparse matrix pattern.

In summary, a kernel’s position within our RLM extension depends not only on the computing hardware platform (the FPGA) and the (block) computer solver, as is typically the case in RLM, but also on the non-zero coefficient pattern of the matrix.

### 3.3. Extending the Proposal to Address Energy Consumption

**Figure 4:**
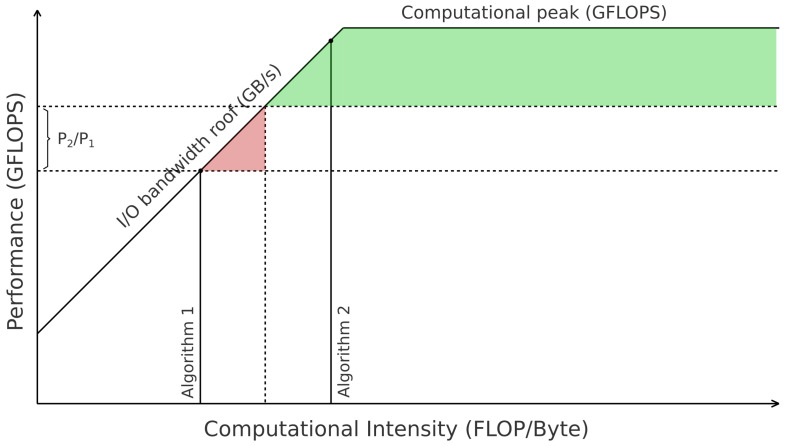
Extension of RLM for runtime and energy consumption optimization.

To evaluate the energy consumption, we include more information in our extension of the RLM. We draw a line parallel to the X-axis corresponding to the peak of performance of the *Scalar* method. Then, we draw another parallel line corresponding to the performance that matches the energy consumption of the *Scalar* method with the power of the *Block* method. In other words, if $P_{1}$ is the average power of the *Scalar* method, $P_{2}$ is the average power of the *Block* method, and flop is the number of required floating-point operations, then the energy spent in the two lines is $E=P_{1}t_{1}=P_{2}t_{2}$. Therefore, $t_{2}=\left(P_{1}/P_{2}\right)t_{1}$, and the second line is drawn at ${\mathrm{perf}}_{2}=\mathrm{flop}/t_{2}=\left(P_{2}/P_{1}\right)\left(\mathrm{flop}/t_{1}\right)=\left(P_{2}/P_{1}\right){\mathrm{perf}}_{1}$. This defines two zones: the first where the *Block* algorithm decreases the runtime but increases the energy consumption, and the second, where both metrics are improved. Furthermore, the graph shows the minimum CIA for improving energy consumption. Figure 4 summarizes the idea, where the first zone is red and the second is green.

## 4. Experimental Evaluation

In this section, we present the experiments carried out to evaluate the performance of our kernels. First, we describe our computing platforms and the equipment used to evaluate runtime and power consumption. Next, we present the test cases and the numerical results, accompanied by the corresponding analysis. Last, we analyze the results using the extended RLM variant considering energy consumption.

### 4.1. Experimental Setup

We used the Alveo U50 board from AMD-Xilinx (San Jose, CA, USA) for the experimental evaluation, an acceleration platform for data center applications based on an FPGA with Ultrascale+ architecture. This board includes 8 GB of built-in high bandwidth memory DRAM and QSFP28 connections for 100 GbE applications. Its maximum power consumption is 75 W, which, added to the high computing capacities and memory bandwidth, achieves excellent energy efficiency. It has 872 K logic elements, 1743 K registers, 28 MB of internal RAM, and 5952 DSP blocks. We compiled the cores for the Alveo platform using Xilinx Vitis 2020.2. For comparison purposes, we have included a traditional CPU system with two Intel Xeon Silver 4208 processors with eight cores each and 80 GB of RAM.

We measured the power consumption as follows:

- In the Alveo U50, we use the board’s internal sensors that provide current, voltage, and temperature readings while the kernel is running. The driver Xilinx Runtime (XRT) sends these values to the host.
- For the Intel processor, we measure CPU and memory consumption using RAPL (which estimates the dissipated power based on performance counters and a device power model).

We obtain the execution times through the operating system libraries in the case of the Intel processor and OpenCL profiling functions in the FPGA. The runtime measurements are the average of multiple iterations of the cores. In the case of the power measurements, the measured power is the average of the readings collected within 3 min of execution, with a warm-up time of 2 min.

### 4.2. Test Cases

We validate the proposal using matrices from the SuiteSparse Matrix Collection (formerly known as UF Sparse Matrix Collection). We choose matrices with diverse characteristics and non-zero patterns, with dimensions (*n*) from 17,000 to 40,000 and a number of non-zeros (nnz) between 14,765 and 16,171,169. Table 1 shows the main characteristics of the matrices.

### 4.3. Experimental Results

This section summarizes the experimental results for the *Scalar* and *Block* methods. For the *Block* kernel, we used block size 16 × 16. For comparison purposes, we include the results for the CPU running MKL implementation of SpMv. Table 2 includes the runtime and energy consumption of the three variants. The results show that the new *Block* algorithm, which uses fine-grained parallelism, significantly reduces the execution times of the *Scalar* version in most cases. Another noteworthy aspect is that the reductions are more important as nnz increases, reaching a maximum of 7.5×. For some of the smallest matrices, the *Block* method decreases the performance. This is because the sparsity pattern and the number of non-zeros lead to excessive padding. The CPU version outperforms the FPGA methods in all cases.

Regarding the energy consumption, due to the reduced power consumption of the FPGA, for the cases where the execution time between both platforms is comparable (large matrices) the FPGA is more energy efficient. For the two largest matrices, the FPGA is 2× more efficient than the CPU in this aspect.

The execution time on the FPGA is linear with nnz after padding. This does not hold true for the CPU. First, it can be observed that when nnz exceeds the cache level, there is a relative increase in execution time. On the other hand, there are some cases in which matrices with a higher nnz run faster than those with fewer non-zeros. Examples of this are qpband vs. ex9, ted_B vs. nasa2910, and TS300 vs. nd6k. It is not easy to determine a cause for this because we do not have access to the kernel that executes MKL. However, it can be observed that in these cases, the matrices that execute faster, even though they have a higher nnz, have smaller dimensions (rows and columns). On the FPGA side, if we compare nd6k with TS300, we can see that the first has more padding than the second, directly affecting the CIA and the achieved GFLOPs. This and the above explain why the TS300 matrix is more energy efficient on the FPGA, whereas the nd6k matrix, despite having more non-zeros, is more efficient on the CPU.

**Figure 5:**
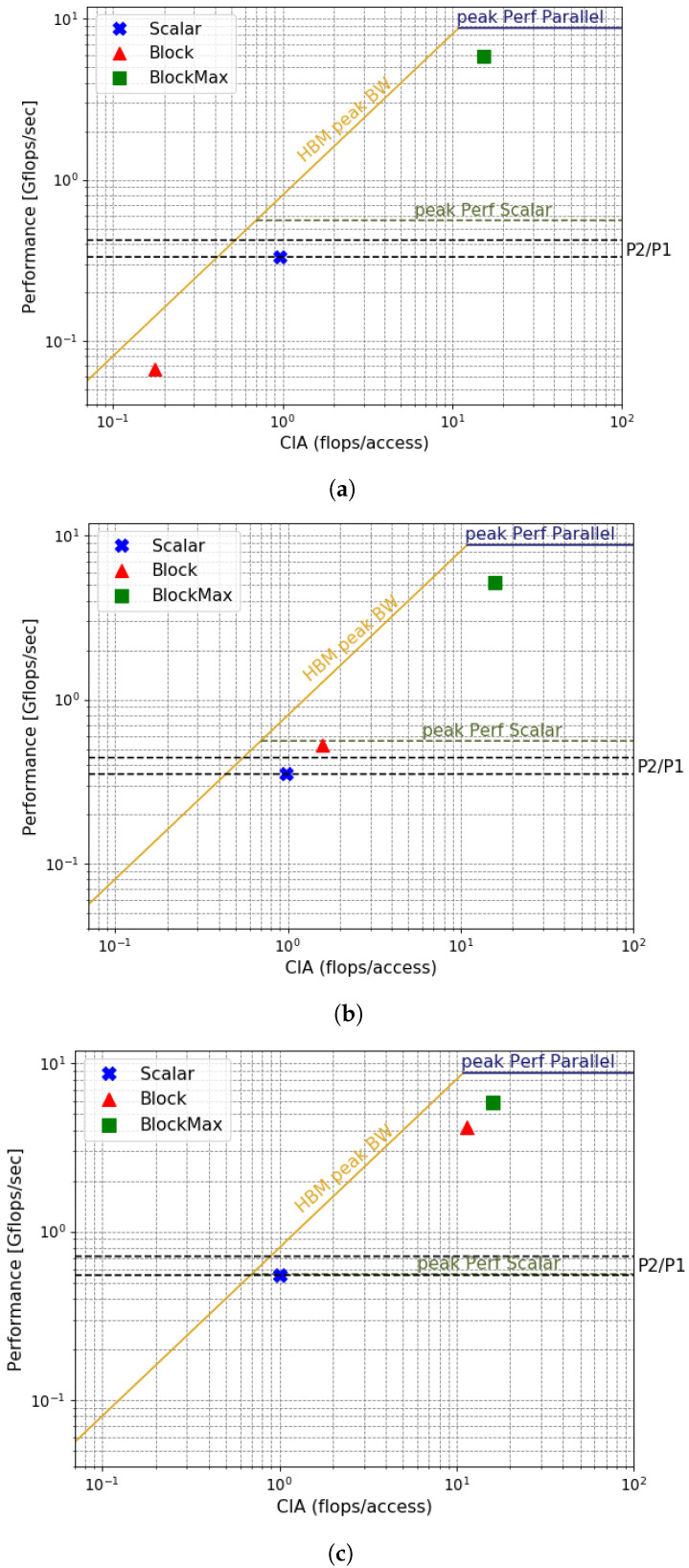
Roofline model of three different matrices: (**a**) chipC0, (**b**) gyro_k, and (**c**) TS2383. P2 represents the power consumption of the *Block* version and P1 the power of the *Scalar* version.

We selected three matrices as an example for the roofline analysis between the two FPGA variants: chipC0, gyro_k, and TS2383. These cases represent three distinct scenarios. In the first one, the *Block* version drastically reduces the performance; for the second one, both methods yield similar runtimes; and for the third one, the *Block* method greatly increases performance. In Figure 5, we show the RLM graph for the *Scalar* (blue) and *Block* (red) results of the three matrices. We include, in green, the maximum attainable performance of the *Block* kernel (*BlockMax*). This represents the attainable performance of the *Block* method when all the matrix elements are taken into account, including the added zeros.

We obtain the peak performance roofs for each method as the maximum number of FLOPs that the kernel architecture can attain, considering the number of parallel floating-point operations and the clock frequency. The kernels approach the performance roof when the average zero-padding per block tends to zero. For the memory access roof (measured in memory access per second), we first measure the maximum bandwidth (in GB/s) of one HBM channel with an interface of 512 bits using a kernel specifically designed for that purpose. Then, we convert this measure into accesses per second by dividing by the number of bytes of one access. Finally, we adjust the value to consider the number of channels used in the kernel.

This example illustrates the benefits of our proposal. By calculating the CIA for a specific matrix and block size, we can evaluate the effectiveness of the *Block* version without executing the kernel. To achieve this, we draw a vertical line on the computed CIA and determine the attainable performance by intersecting this line with the corresponding roof of the RLM. This approach offers an analytical tool for predicting the runtime and energy consumption behavior of a kernel for each matrix, eliminating the necessity for the time-consuming process of compiling and running the design on an FPGA.

**Figure 6:**
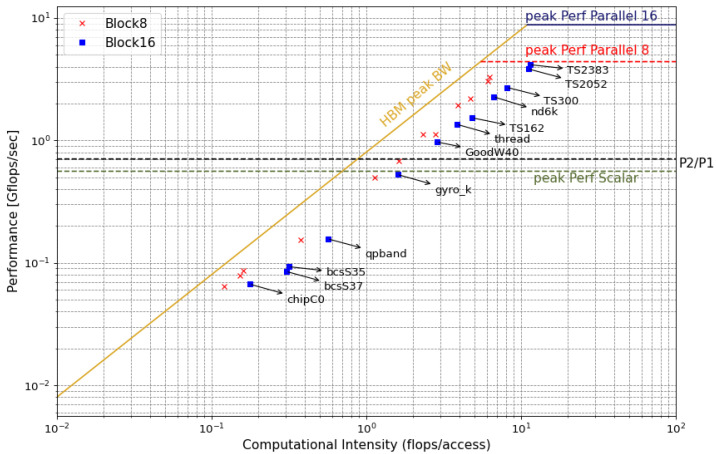
Roofline model for several matrices using block size of 8 (red cross) and 16 (blue square).

In the same line, Figure 6 summarizes the behavior of the SpMv kernel for various matrices using Blocked-ELL with block sizes of 8 × 8 and 16 × 16. In these cases, the utilization of 16 × 16 blocks outperforms their 8 × 8 counterpart, as they attain higher values of CIA in the memory-bound zone of the RLM. Furthermore, with the assistance of the RLM, it becomes straightforward to identify which matrices benefit from blocked kernels and which are better suited for the Scalar version. It can be observed that the points situated below the *peak Perf Scalar* line will not experience a performance improvement compared to the *Scalar* version, resulting in poorer energy performance (see Section 3.3). Conversely, points positioned above the P2/P1 line demonstrate a noticeable performance improvement, leading to a reduction in energy consumption.

## 5. Concluding Remarks

In this paper, we have proposed an extension of the roofline model for FPGAs intended to optimize sparse linear algebra kernels based on blocked representation formats, both from the runtime and energy consumption perspectives. In particular, we implemented a SpMv kernel that leverages a block-sparse storage format to attain concurrency in the data access and parallelism in the computations. The experimental evaluation of our methods, performed on a cutting-edge FGPA, confirms the benefits of this kind of block format for sparse algebra. In the same line, the proposed use of the RLM allows the selection of the best option (between the *Scalar* algorithm and the *Block* algorithm with different block dimensions) to improve the runtime and energy consumption effortlessly.

We plan to address several lines in future work. As the first step, we plan to include specific optimizations on our kernel to improve the performance. Secondly, we expect to extend our ideas to other sparse NLA kernels, such as the SpMM, SpTrSv, and SpGEMM, evaluating the usefulness of our RLM extension. Finally, sparse matrix reordering techniques can be applied to understand the impact of the computational intensity on our kernels’ performance.

## Figures and Tables

**Table 1:** Evaluated matrices, with number of rows (*n*) and non-zero elements (nnz). The last column is the average density of the blocks resulting from the Blocked-ELL format for block size 16×16.

Matrix	*n*	nnz	Avg. Density of Blocks (%)
bcsS37	25,503	14,765	4
bcsS35	30,237	18,211	4
qpband	20,000	30,000	5
ex9	3363	51,417	21
body4	17,546	69,742	6
body6	19,366	77,057	6
ted_B	10,605	77,592	20
ted_B_un	10,605	77,592	20
nasa2910	2910	88,603	24
s3rmt3m3	5357	106,526	27
s2rmq4m1	5489	143,300	34
chipC0	20,082	281,150	1
cbuckle	13,681	345,098	31
olafu	16,146	515,651	33
gyro_k	17,361	519,260	10
GoodW40	17,922	561,677	18
bcsstk36	23,052	583,096	27
msc23052	23,052	588,933	13
msc10848	10,848	620,313	18
raefsky4	19,779	674,195	27
TS162	20,374	812,749	31
nd3k	9000	1,644,345	43
thread	29,736	2,249,892	24
TS300	28,338	2,943,887	51
nd6k	18,000	3,457,658	42
TS2052	25,626	6,761,100	70
TS2383	38,120	16,171,169	71

**Table 2:** Runtime and energy results for CPU and FPGA implementations.

Matrix	$t_{\mathrm{CPU}}$ MKL (ms)	$t_{\mathrm{FPGA}}$ *Scalar* (ms)	$t_{\mathrm{FPGA}}$ *Block* (ms)	$E_{\mathrm{CPU}}$ MKL (mJ)	$E_{\mathrm{FPGA}}$ *Scalar* (mJ)	$E_{\mathrm{FPGA}}$ *Block* (mJ)
bcsS37	0.012	0.280	0.324	0.6	5.2	6.6
bcsS35	0.012	0.331	0.366	0.6	6.2	7.5
qpband	0.017	0.258	0.356	0.8	4.8	7.3
ex9	0.015	0.409	0.195	0.7	7.6	4.2
body4	0.024	0.414	0.698	1.1	7.7	14.9
body6	0.025	0.434	0.791	1.2	8.1	16.9
ted_B	0.031	0.625	0.285	1.5	11.7	6.1
ted_B_un	0.037	0.686	0.297	1.7	12.8	6.3
nasa2910	0.022	0.890	0.243	1.0	16.6	5.2
s3rmt3m3	0.026	0.595	0.259	1.2	11.1	5.5
s2rmq4m1	0.028	0.686	0.273	1.2	12.8	5.8
chipC0	0.067	1.576	7.772	3.1	29.4	167.1
cbuckle	0.073	1.775	0.738	3.2	33.1	15.7
olafu	0.117	2.527	1.008	5.3	47.1	21.5
gyro_k	0.097	2.754	1.837	4.7	51.4	39.2
GoodW40	0.104	4.064	1.078	5.0	75.9	23.1
bcsstk36	0.130	3.014	1.240	6.1	56.2	26.5
msc23052	0.183	3.052	2.097	8.5	56.9	44.7
msc10848	0.181	3.546	1.703	8.2	66.1	36.3
raefsky4	0.125	3.097	1.369	6.3	57.7	29.2
TS162	0.216	3.140	0.991	10.4	58.6	21.0
nd3k	0.322	6.755	1.841	15.9	126.0	39.3
thread	0.448	11.120	3.102	23.5	207.9	67.5
TS300	0.986	10.290	2.024	49.3	192.3	43.7
nd6k	0.761	13.962	2.839	40.1	261.4	62.3
TS2052	2.839	23.093	3.284	139.4	431.2	72.1
TS2383	6.411	54.543	7.253	323.7	1019.5	159.5

## Data Availability

Not applicable.
